# Investigation Using Granular Material Obtained from High-Density Polyethylene and Polypropylene Plastic Waste as Aggregate in Cementitious Systems

**DOI:** 10.3390/polym18040542

**Published:** 2026-02-23

**Authors:** Şemsi Yazıcı, Göksu Pılsım, Hatice Gizem Şahin, Demet Yavuz, Ali Mardani

**Affiliations:** 1 Civil Engineering Department, Ege University, İzmir 35100, Türkiye; semsi.yazici@ege.edu.tr (Ş.Y.); goksuplsm@gmail.com (G.P.); 2 Civil Engineering Department, Bursa Uludag University, Nilüfer-Bursa 16059, Türkiye; haticegizemsahin@gmail.com; 3 Civil Engineering Department, Yüzüncü Yıl University, Van 65080, Türkiye; demetyavuz@yyu.edu.tr

**Keywords:** high-density polyethylene, polypropylene, waste management, plastic-waste aggregate, workability

## Abstract

The accumulation of plastic waste represents a significant environmental challenge worldwide, and its reuse in construction materials offers a sustainable management alternative. This study investigates the use of recycled high-density polyethylene (HDPE) and polypropylene (PP) granules as partial volumetric replacements (10%, 20%, and 30%) for limestone aggregate in mortar mixtures. A total of seven mixtures were produced and evaluated in terms of flow value, unit weight, water absorption, porosity, compressive strength, flexural strength, and capillary water absorption. In comparison to the control mixture, it was found that the use of plastic aggregate improved the workability. It was found that the flexural and compressive strengths of mixtures decrease when plastic aggregate is added. Additionally, it was understood that utilization of plastic aggregate in mixtures caused an increase in water absorption rate and porosity values. HDPE and PP plastic aggregates increased flow by 9% to 13% and reduced unit weight by 15 to 15.3%, while compressive and flexural strengths decreased by 48 to 30% and 46 to 54%, respectively. The optimum replacement level was 10% for both HDPE and PP mixtures.

## 1. Introduction

Environmental pollution, one of the biggest problems in the world, continues to increase. It has been reported that one of the main causes of this situation is plastic waste [1,2,3]. Plastic wastes require hundreds of years to decompose in nature and, during this process, release harmful greenhouse gases such as methane and ethylene, which accelerate global warming and pose significant threats to living organisms [4,5]. Plastics are lightweight materials produced through chemical transformations using petroleum-based products and natural gas as raw materials [6,7]. They do not rust or corrode and have become indispensable due to their flexibility, durability, ease of shaping, and high thermal and electrical insulation properties. Furthermore, when the processing temperature of steel (approximately 1400 °C) is compared with that of plastics (around 250 °C), it becomes evident that plastics can be processed much more easily and with significantly lower energy consumption [8].

The disposal of industrial and domestic waste has become a major global challenge [9,10,11,12], and one of the most effective approaches to this problem is the reuse of such materials. In this context, increasing attention has been given to the potential use of waste plastics in concrete and mortar production [13,14]. Plastics are commonly classified into six main groups: polyethylene terephthalate (PET), high-density polyethylene (HDPE), polyvinyl chloride (PVC), low-density polyethylene (LDPE), polypropylene (PP), and polystyrene (PS). In previous studies, waste plastics have been incorporated into cementitious systems either as aggregates or fibers [15,16], with the dual aim of mitigating environmental problems and improving the properties of concrete and mortar composites. At this point, the aim is to find a partial solution to a major environmental problem and to improve the properties of concrete and mortars. Numerous investigations on the impact of waste plastics on cementitious system performance have been published in the literature [17,18,19,20]. It has been reported that when used as fibers, waste plastics are generally added at rates not exceeding 1% by volume [17,18,19]. Although plastic fibers may improve mechanical performance, they typically reduce the workability of concrete. Additionally, several studies have explored the use of waste plastics as aggregates to enable higher incorporation rates in mixtures [21,22]. Ahmad et al. [23] investigated the usability of plastic wastes as aggregates and fibers in cement and mortars. Slump, compressive strength (CS), flexural strength (FS), tensile strength, drying shrinkage, water absorption, and porosity rate tests were carried out on the prepared samples. According to the test results, it was seen that using plastic waste as fiber increased the mechanical performance, but using it as aggregate decreased the performance of the concrete. Al-Mansour et al. [24] investigated the interaction between the matrix and plastic aggregates—specifically ethylene vinyl acetate (EVA), polypropylene (PP), and polyamide (PA)—in cement mortars. Density, FS, CS, and ductility were measured. Their findings revealed increased void content and reduced density, FS, and CS, alongside enhanced deformation capacity and energy absorption with increasing EVA content. Beller [25] examined the behavior of mortars containing PET aggregates at high temperatures. Six mixtures containing 0–30% PET by volume were exposed to temperatures between 100 and 400 °C for varying durations. Higher PET contents led to reductions in consistency, unit weight (UW), ultrasound pulse velocity, CS, and FS. Gregorova et al. [26] investigated the production of lightweight concrete using cable, polystyrene, and ethylene vinyl acetate (EVA) waste as aggregates. The plastic waste was produced in nine different combinations: 100%, 75%/25%, and 50%/50%. Mixtures containing 100% polystyrene achieved the best thermotechnical properties and had the lowest bulk density values. Increasing the EVA waste content resulted in increased thermal conductivity values as well as strength properties. Saikia and Brito [21] studied the usability of waste PET in concrete. Experiments were carried out on concrete samples produced by replacing the aggregate with PET aggregates of three different shapes and sizes at 5%, 10%, and 15% by volume. With the increase in the use and ratio of PET aggregate, the slump value, FS and CS, and elasticity modulus of the concrete decreased, but the abrasion resistance increased. Aiello et al. [22] investigated the usability of waste tire rubber pieces in concrete mixtures by replacing natural aggregate. According to the results of their study, it was observed that the workability increased, the UW decreased, and the FS and CS decreased in the rubber substituted samples. Industrial challenges must be overcome in order to increase the widespread use of plastic aggregates. In studies conducted in the literature, various methods such as mechanical, physical, coating, thermal, chemical, and radiation methods have been suggested to ensure the adhesion of plastic to concrete, as stated in a study by Abu-Saleem et al. [27]. In this study, mechanical processing was preferred because it does not alter the chemical structure of the plastic. Mechanical processing comprises a series of physical operations aimed at size reduction and material fractionation. The process typically includes the separation of different material types [28], fragmentation into smaller pieces [29], and further particle size reduction through grinding [27,30].

It was previously emphasized that significant ecological gains can be achieved by using waste plastics in cementitious systems, re-evaluating wastes and producing cement-based composites with lower CO_2_ content. In this study, granular products produced by recycling HDPE and PP wastes were used instead of aggregate at 10%, 20%, and 30% by volume. The mechanical and physical properties of the produced plastic granulated mortars were investigated by performing tests on flowability, unit volume weight, water absorption and porosity ratio, FS–CS, and capillary water absorption.

## 2. Materials and Methods

### 2.1. Materials

#### 2.1.1. Cement

Portland cement, CEM I 42.5 R, was employed as a binder. Cement XRF results are shown in Table 1.

#### 2.1.2. Aggregate

##### Limestone Aggregate

**Figure 1:**
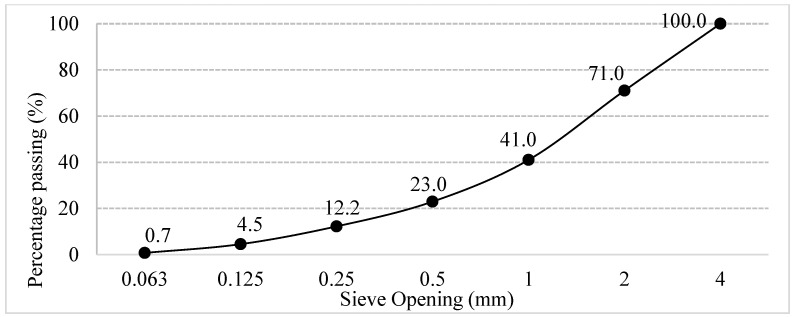
Gradation curve of limestone aggregate.

In the study, 0–4 mm sized fine aggregate of limestone origin was used. Specific gravity and UW of the aggregate are given in Table 2. Gradation of the aggregate is given in Figure 1.

##### HDPE and PP Aggregate

**Figure 2:**
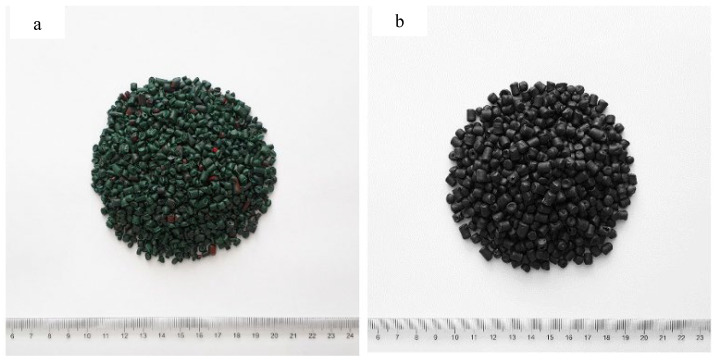
Representation of (**a**) HDPE and (**b**) PP granules.

**Figure 3:**
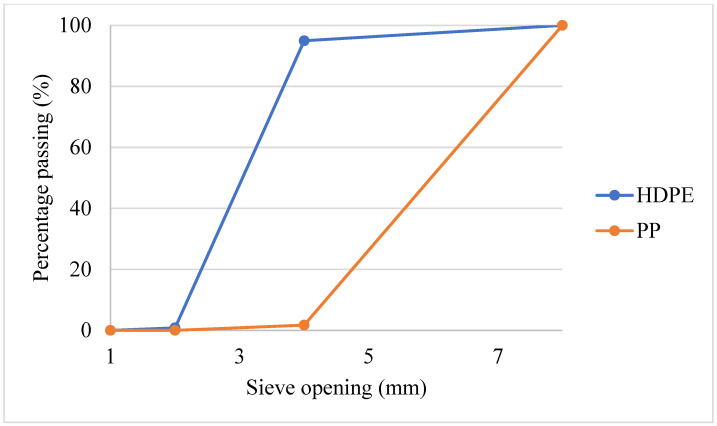
Gradation curve of HDPE and PP granules.

Granules produced from recycled HDPE and PP used as plastic aggregates are shown in Figure 2a,b. HDPE granules were obtained by cutting clean waste buttermilk bottles. PP granules were obtained by cutting clean waste ice cream boxes. HDPE and PP were used in mortar mixtures by replacing fine aggregate at rates of 10%, 20%, and 30% by volume. The specific gravity and UW of HDPE and PP aggregate are given in Table 2, and the gradation curves are given in Figure 3.

### 2.2. Mixing Proportions and Preparation of Mixtures

In this study, HDPE and PP type recycled plastic granules were tested by substituting 10%, 20%, and 30% of the fine aggregate volume. The amounts of materials used in 1 m^3^ mortar mixtures are shown in Table 3. Each mixture in the study was prepared in accordance with the EN 196-1 Standard. The naming of the mixtures was done according to the type of plastic aggregate used and the usage rate. The mixture in which 10% HDPE granular aggregate is used is called HDPE-10, while the mixture in which 20% PP granular aggregate is used is called PP-20.

### 2.3. Method

Flow table and UW tests were carried out on the mixtures produced within the scope of the study. For all mixtures, 7-, 28-, 56-, and 90-day FS and CS tests were performed, and the ideal plastic aggregate usage rate was determined according to the results obtained from these tests. Water absorption, porosity, and capillary water absorption tests were performed on the mixtures with this determined ratio.

In this study, a series of standardized tests were conducted to determine the fresh and hardened properties of the mixtures. The flow value of all mixtures was determined by the flow table test in accordance with EN 1015-3. Unit weight (UW) tests were performed for all mixtures, while water absorption and apparent porosity were determined according to ASTM C642. Water absorption and porosity tests were conducted on 28-day specimens containing 10% plastic aggregate substitution, which was identified as the optimum mixture ratio based on the flexural and compressive strength results.

The mechanical properties were evaluated through compressive strength and flexural strength tests in accordance with EN 196-1. Compressive strength tests were carried out on 40 × 40 × 40 mm cube specimens at curing ages of 7, 28, 56, and 90 days. Flexural strength tests were performed on 40 × 40 × 160 mm prismatic specimens at 7, 28, 56, and 90 days. Each measurement was repeated three times.

Capillary water absorption behavior was determined in accordance with ASTM C1585. The weights of the samples placed on the tray, adjusted to have an even water surface, were measured at 1, 5, 10, 20, 30, 60, 120, 180, 240, 300, and 360 min for the primary capillary water absorption test, and at 1, 2, 3, 4, 6, 7, and 8 days for the secondary capillary water absorption test. Graphs of these measurements were plotted against time, and the primary and secondary capillary water absorption coefficients were calculated. Each measurement was repeated three times.

Capillary water absorption values of the samples were calculated with the following formula:

$$I=\frac{m_{t}}{a*d}$$

*I*: capillary water absorption (mm);

$m_{t}$: change in sample mass at time t (g);

a: surface area of sample in contact with water (mm^2^);

d: density of water (g/mm^3^).

## 3. Results and Discussion

### 3.1. Fresh State Properties

#### Flowability Experiment

**Figure 4:**
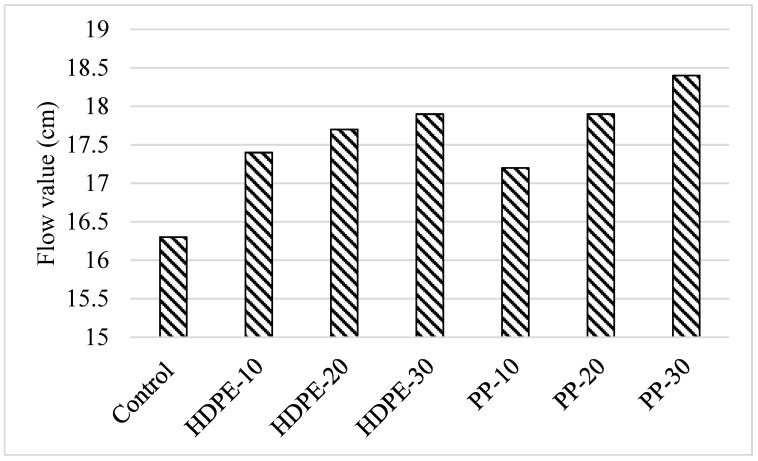
Flow values of the mixtures.

Figure 4 displays the results of the dispersion tests conducted on the mixes created during the study. Regardless of the plastic aggregate type and substitution rate, the addition of waste plastic aggregate to the control mixture caused the flow values to increase between 6% and 12%. Regardless of the type of plastic aggregate, the increase in the flow value of the mixtures was more dominant with the increase in the plastic aggregate substitution rate. When the effect of the type of waste plastic aggregate used in the mixtures on the flow value was examined, it was determined that the use of 10%, 20%, and 30% of HDPE granular aggregate in mortar mixtures increased the flow value by 6%, 8%, and 9%, respectively, compared to the control mixture. This increase was measured as 5%, 9%, and 13% in mixtures containing PP aggregate, respectively. This increase is thought to be due to the lower water absorption capacity of plastic aggregate compared to limestone aggregate and the decrease in surface area due to the increase in the average aggregate size with its smooth surface. Al-Manaseer et al. [31] reported that this increase was attributed to the non-absorbent characteristic of PP, causing more free water to form in concrete mixtures. It was stated that fluidity was improved with the increase in free water content. Nonetheless, a number of researchers have noted that the plastic particle form also affects how workable fresh concrete is, and that if the particles are sharply edged, the flow value may drop [21,32]. As is known, fluidity increases with the addition of particles with spherical edges [21]. In a study by Yang et al. [33], the effect of using 10, 15, 20, and 30% recycled modified PP fiber instead of sand on the workability of self-compacting lightweight concrete was investigated. As a result, flow value increased with the increase in plastic content. However, fresh concrete had a tendency to bleed when the plastic aggregate substitution rate reached 30%. It was, therefore, suggested that the water-to-binder ratio and water content should be changed if more sand is substituted with plastic particles. Safi et al. [34] and Ahmad et al. [23] also reported findings that were comparable.

### 3.2. Hardened State Properties

#### 3.2.1. Compressive Strength

The 7-, 28-, 56-, and 90-day CS results are shown in Table 4. When the CS results were examined, it was understood that the use of plastic aggregate caused a decrease in CS values compared to the control sample, regardless of the plastic aggregate type and substitution rate. Regardless of the sample age, the mixture with the highest CS value compared to the control mixture was PP-10, and that with the lowest CS value was HDPE-30. In a study conducted by Awad et al. [35], it was determined that PP provides higher strength than HDPE. Contrary to this result, Rahman and Nurdiana [36] stated that HDPE plastic wastes are stronger, harder, denser, and more resistant to high temperatures compared to other plastics.

**Figure 5:**
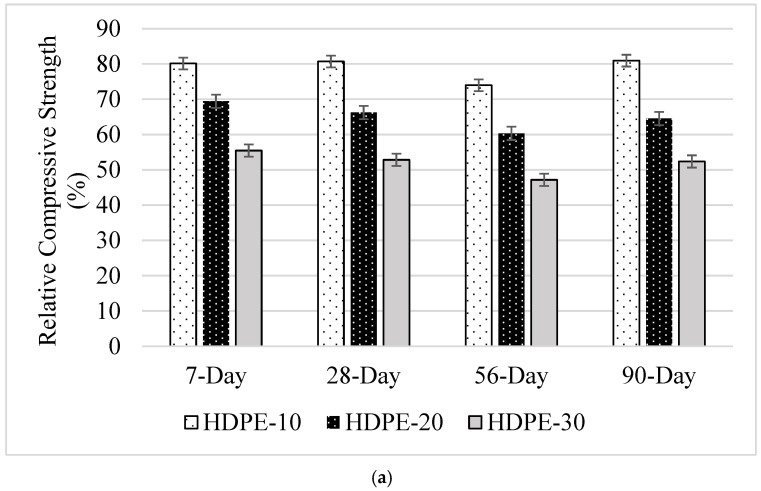

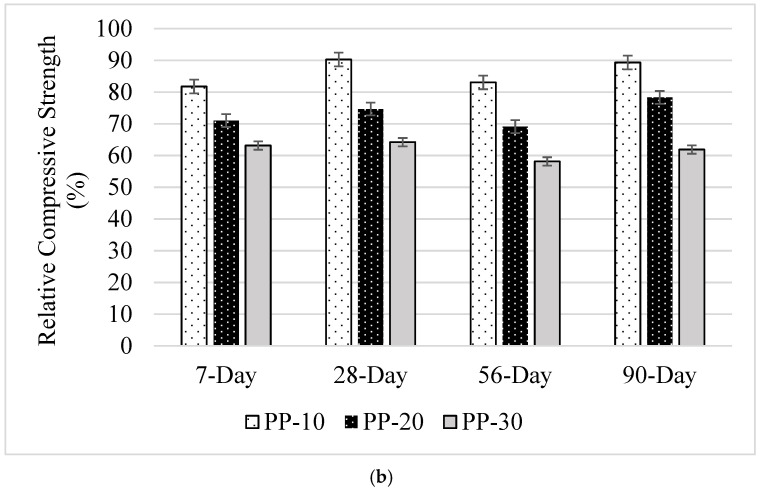
Relative CS values of samples containing (**a**) HDPE and (**b**) PP aggregates compared to the control mixture.

Relative strength values were plotted according to the control mixture (Figure 5a,b). When the CS of the samples containing HDPE granules and having a substitution rate of 10%, 20%, and 30% were compared with the control sample (Figure 5a), 7-day CS decreased by 20%, 31%, and 45%, 28-day CS decreased by 19%, 34%, and 47%, 56-day CS decreased by 26%, 40%, and 53%, and 90-day CS decreased by 19%, 35%, and 48%, respectively. The highest CS of the samples containing HDPE plastic aggregate at 7, 28, 56, and 90 days were achieved in HDPE-10 samples. The strength values decreased due to the increase in aggregate replacement ratio in the mixtures, and the lowest CS value was obtained in the HDPE-30 sample, regardless of the sample age.

When the relative CS values of the samples in which PP granules were used as aggregate were examined (Figure 5b), it was understood that the effect of the increase in the plastic aggregate usage rate on the CS value of the mixtures was similar to that of HDPE aggregate. It was determined that the highest CS value was obtained when the PP usage rate was 10%, and the lowest strength values were obtained when it was 30%. It is thought that the decrease in CS in mixtures with the increase in the use of plastic aggregate and its usage rate is due to the smooth structure of the plastic aggregate surface and the weakening in the interfacial transition zone (ITZ). In a study by Belmokaddem et al. [37], it was reported that plastic aggregates such as HDPE have a lower dynamic elastic modulus and that the adhesion between the polymer and the cement matrix is low due to the difference between the elastic modulus of the polymer particles and the elastic modulus of the surrounding cement paste. Similar statements have been made by various researchers [38]. According to Jones and Facaroau [39], partial replacement of fine aggregate with plastic aggregate such as PET leads to a gradual decrease in modulus because PET is less resistant than aggregate and deforms less when an equivalent stress is applied [38].

Similarly, studies in the literature have reported that the use of this plastic aggregate causes a decrease in the strength values of the mixtures, which may be due to the weak bond between the cement paste and plastic waste or the low strength of these plastic wastes [34,35,36,37,38,39,40,41].

Gavela et al. [42] conducted the usability of PET and PP as aggregates in concrete was investigated. PET and PP were used at 20% and 30% rates instead of normal aggregate. In the experiments, 7- and 28-day CS and FS tests were performed on samples containing the same proportions of plastics, and it was observed that similar results were obtained, but the CS and FS decreased as the plastic ratio in the mixture increased. It was concluded by the researchers that the type of plastic does not affect the strength. Koide et al. [43] investigated the use of a plastic consisting of 85% PET, 15% PE, and PP as lightweight concrete aggregate in concrete. According to the research results, it was seen that this plastic can be used as lightweight concrete aggregate. Although it is observed that the strength decreases with the increase in the plastic ratio in the mixture, it is recommended that the mixing ratio of plastic coarse aggregate by volume should be between 0.35 and 0.40 for both the strength and the density of the concrete.

#### 3.2.2. Flexural Strength

The 7-, 28-, 56-, and 90-day FS results of the mixtures study are shown in Table 5. Similar to the CS results, the use of plastic aggregate caused a decrease in FS values compared to the control sample, regardless of the aggregate type and substitution rate. However, the decrease in the FS values of the mixtures was more severe compared to the CS. This situation is thought to be due to the fact that the use of plastic aggregate, which has a smoother structure compared to limestone aggregate, reduces the cement paste–aggregate adhesion, and the FS test is more sensitive to microcracks in the ITZ region, as mentioned before [44,45]. Similarly, in a study conducted by Safi et al. [30], it was determined that the FS of the mixtures decreased with the increase in the plastic aggregate usage rate. This situation is due to the low resistance of waste materials. Numerous researchers have made similar claims [46,47,48].

HDPE-10 exhibited the highest flexural strength compared to the control mixture. This is thought to be due to the fact that HDPE plastic waste is stronger, harder, and denser than other plastics [36].

**Figure 6:**
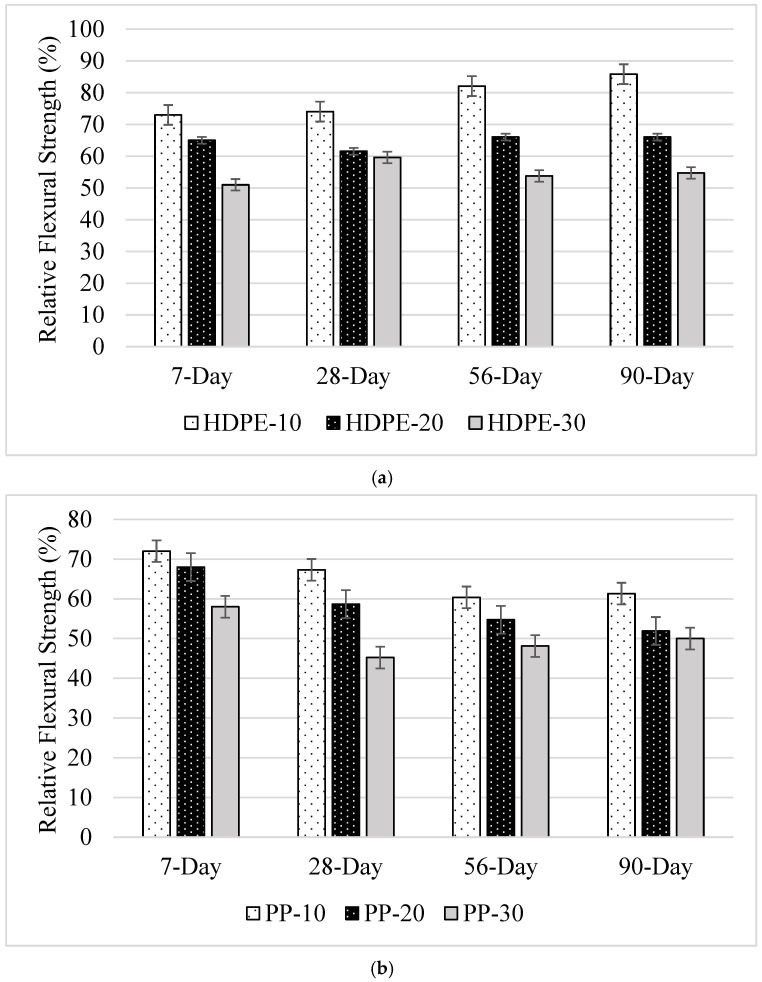
Relative FS values of samples containing (**a**) HDPE and (**b**) PP aggregate compared to the control mixture.

When the FS of the samples with HDPE aggregate and plastic aggregate usage rates of 10%, 20%, and 30% were compared with the control sample (Figure 6b), 7-day FS decreased by 27%, 35%, and 49%, 28-day FS decreased by 26%, 38%, and 40%, 56-day FS decreased by 18%, 34%, and 46%, and 90-day FS decreased by 14%, 34%, and 45%, respectively. The highest 7-, 28-, 56-, and 90-day FS of samples containing HDPE aggregate were obtained in samples with 10% plastic aggregate usage amount. When the relative FS values of the samples in which PP granules were used as aggregate were examined, it was understood that the effect of the increase in the plastic aggregate usage rate on the FS value of the mixtures was similar to that of HDPE aggregate. The highest FS value was obtained when the PP usage rate was 10%, and the lowest FS values were obtained when it was 30%.

#### 3.2.3. UW

Saturated surface dry UW of mixtures containing plastic aggregate are given in Table 6. It was determined that the UW value of the mixtures decreased with the addition of waste plastic aggregate to the mortar mixtures, regardless of the plastic aggregate type and sample age, and this decrease was more pronounced with the increase in the usage rate. When saturated surface dry UW were examined, it was measured that the highest decreases in 7-, 28-, 56-, and 90-day UW weights of the samples compared to the control sample occurred at rates of 15%, 14%, 15%, and 15%, respectively, and these decreases were in samples containing 30% waste PP aggregate. This is due to the fact that plastic aggregates used in mortar mixtures have lower specific gravity values compared to limestone aggregates.

#### 3.2.4. Water Absorption, Porosity, and Capillary Water Absorption

**Figure 7:**
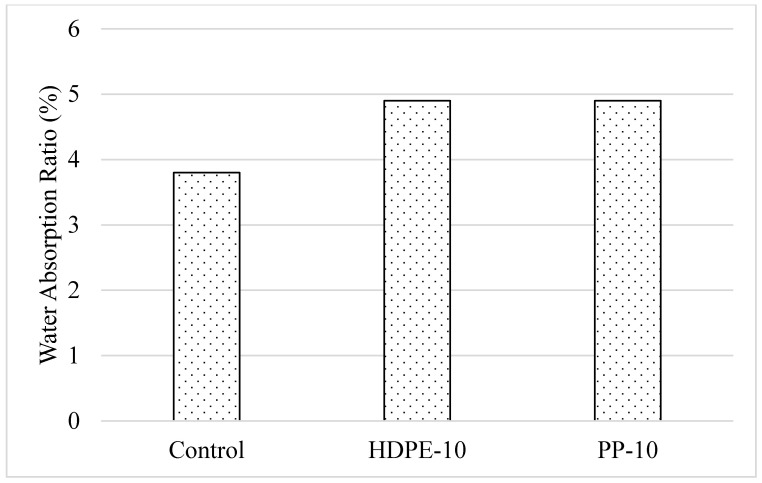
Water absorption ratio of mixtures.

**Figure 8:**
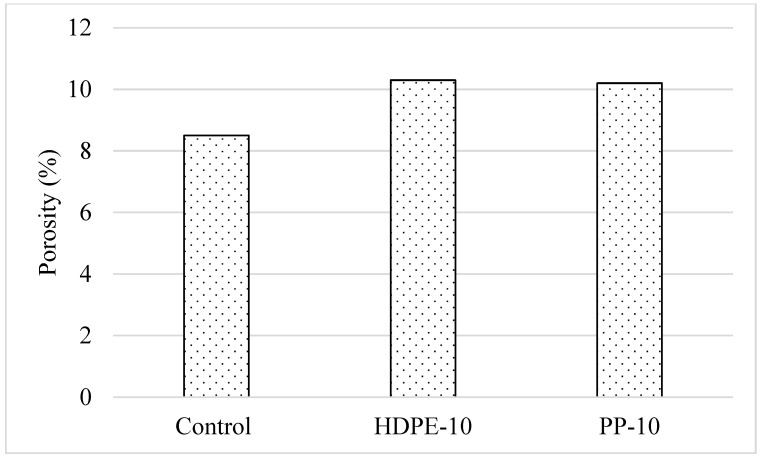
Porosity value of the mixtures.

When the CS and FS state test results were evaluated, it was determined that the optimum plastic aggregate usage rate was generally 10%. For this reason, water absorption, porosity, and capillary water absorption values of the mixtures were investigated on mixtures containing 10% plastic aggregate. Water absorption and porosity rates of 28-day-old samples containing 10% plastic aggregate by aggregate volume are given in Figure 7 and Figure 8, respectively. It was understood that the use of plastic aggregate in mortar mixtures causes an increase of approximately 20% in the water absorption and porosity values of the mixtures, regardless of the aggregate type and usage rate. It was determined that the use of HDPE and PP granules instead of aggregates had similar effects on the water absorption and porosity values of the mixtures and that these parameters were not greatly affected by the type of plastic aggregate. The increase in the porosity in the samples due to the replacement of plastic aggregate with fine aggregate, due to its lower density compared to fine aggregate and the weak interactions of plastic aggregate with the binder, caused an increase in the water absorption rate and porosity value of the mixtures. Similar results have been expressed by various researchers [23,24].

The capillarity coefficients calculated as a result of the capillary water absorption test performed when the mixtures selected within the scope of the study reached 28 days of age are shown in Table 7. As expected, similar to the water absorption results, it was determined that there was an increase in the primary and secondary capillary water absorption values and water penetration depths of the samples containing plastic aggregate compared to the control sample. In comparison to the control, the primary capillary water absorption values in the HDPE-10 and PP-10 samples increased by 332% and 236%, respectively, while the secondary capillary water absorption values increased by 140% and 110%, respectively. This rise in capillary water absorption values is thought to be a result of the weak bond formed with the binder together with the smooth surface of the plastic aggregate.

## 4. Conclusions

In light of the experiments conducted on utilization plastic aggregate in mortars, the following results were obtained.

This study assessed the viability of using HDPE and PP plastic aggregates as partial substitutes in mortar mixtures. The addition of plastic aggregates enhanced workability, increasing flow values by 9–13% while reducing unit weight by approximately 15%, indicating the production of lightweight mixtures. However, a notable decrease in mechanical performance was observed. Both compressive and flexural strengths declined as the plastic content increased, attributed to the lower stiffness of plastic particles and a weaker interfacial transition zone between the cement matrix and the plastic aggregate. Among the ratios investigated, 10% replacement was identified as the optimum level, offering a balance between improved workability and acceptable strength loss. Furthermore, the use of plastic aggregate increased water absorption, porosity, and capillary water absorption. For instance, mixtures with 10% plastic aggregate exhibited higher primary and secondary capillary water absorption values and greater water penetration depths compared to the control. It should be noted that the results are specific to the materials and rates used. Future studies on the durability performance of such mixtures are recommended. Overall, the use of plastic aggregates appears more suitable for non-structural applications.

## Figures and Tables

**Table 1:** Properties of cement.

Oxides		%
Fe_2_O_3_		2.8
CaO		62.4
MgO		1.92
Na_2_O + 0.658 K_2_O		0.82
SiO_2_		18.63
Al_2_O_3_		4.73
SO_3_		3.26
Loss on Ignition		4.39
Na_2_O		0.29
K_2_O		0.81
Cl^−^		
Specific gravity		3.08
Specific surface area (cm^2^/g)		3370
Setting Time (min)	Initial	172
	Final	281

**Table 2:** Properties of aggregates.

Properties	Aggregate Type		
	Limestone Aggregate	HDPE Granules	PP Granules
Compact UW (kg/m^3^)	1693	521	557
Dry bulk specific gravity	2.66	0.94	0.9

**Table 3:** Amount of material for 1 m^3^ mortar mixture (kg).

Mixture	Cement	w/c Ratio	Limestone Aggregate	HDPE Granular Aggregate	PP Granular Aggregate	Theoretical UW (kg/m^3^)
Control	514	0.5	1542	-	-	2313
HDPE-10			1388	54.1	-	2213
HDPE-20			1234	108.3	-	2113
HDPE-30			1079	162.4	-	2012
PP-10			1388	-	51.8	2211
PP-20			1234	-	103.6	2109
PP-30			1079	-	155.5	2006

**Table 4:** CS values of mortar mixtures (MPa).

Mixture	Age (Day)			
	7	28	56	90
Control	49.4	54.5	63.8	60.9
HDPE-10	39.6	44	47.2	49.3
HDPE-20	34.3	36.1	38.5	39.3
HDPE-30	27.4	28.8	30.1	31.9
PP-10	40.4	49.2	53	54.4
PP-20	35.1	40.7	44.1	47.7
PP-30	31.2	35	37.1	37.7

**Table 5:** FS values of mortar mixtures (MPa).

Mixture	Age (Day)			
	7	28	56	90
Control	10	10.4	10.6	10.6
HDPE-10	7.3	7.7	8.7	9.1
HDPE-20	6.5	6.4	7	7
HDPE-30	5.1	6.2	5.7	5.8
PP-10	7.2	7	6.4	6.5
PP-20	6.8	6.1	5.8	5.5
PP-30	5.8	4.7	5.1	5.3

**Table 6:** Saturated surface dry UW values of mortar mixtures (kg/m^3^).

Mixture	Age (Day)			
	7	28	56	90
Control	2379	2383	2402	2398
HDPE-10	2269	2275	2285	2290
HDPE-20	2149	2190	2193	2194
HDPE-30	2021	2051	2063	2065
PP-10	2241	2249	2276	2269
PP-20	2165	2160	2171	2172
PP-30	2016	2040	2035	2038

**Table 7:** Capillarity coefficients of samples containing plastic aggregates.

Mixture	Primary Capillary Water Absorption (mm/sn^0.5^)	Secondary Capillary Water Absorption (mm/sn^0.5^)
Control	0.0025	0.001
HDPE-10	0.0108	0.0024
PP-10	0.0084	0.0021

## Data Availability

All data, models, and code generated or used during the study appear in the submitted article.
